# The Use of Therapeutic Music Training to Remediate Cognitive Impairment Following an Acquired Brain Injury: The Theoretical Basis and a Case Study

**DOI:** 10.3390/healthcare8030327

**Published:** 2020-09-08

**Authors:** Cheryl Jones

**Affiliations:** Department of Music, Wilfrid Laurier University, Waterloo, ON N2L 3C5, Canada; con.brio.piano@gmail.com

**Keywords:** music training, acquired brain injury, cognitive rehabilitation, attention, executive function, memory

## Abstract

Cognitive impairment is the most common sequelae following an acquired brain injury (ABI) and can have profound impact on the life and rehabilitation potential for the individual. The literature demonstrates that music training results in a musician’s increased cognitive control, attention, and executive functioning when compared to non-musicians. Therapeutic Music Training (TMT) is a music therapy model which uses the learning to play an instrument, specifically the piano, to engage and place demands on cognitive networks in order to remediate and improve these processes following an acquired brain injury. The underlying theory for the efficacy of TMT as a cognitive rehabilitation intervention is grounded in the literature of cognition, neuroplasticity, and of the increased attention and cognitive control of musicians. This single-subject case study is an investigation into the potential cognitive benefit of TMT and can be used to inform a future more rigorous study. The participant was an adult male diagnosed with cognitive impairment as a result of a severe brain injury following an automobile accident. Pre- and post-tests used standardized neuropsychological measures of attention: Trail Making A and B, Digit Symbol, and the Brown–Peterson Task. The treatment period was twelve months. The results of Trail Making Test reveal improved attention with a large decrease in test time on both Trail Making A (−26.88 s) and Trail Making B (−20.33 s) when compared to normative data on Trail Making A (−0.96 s) and Trail Making B (−3.86 s). Digit Symbol results did not reveal any gains and indicated a reduction (−2) in free recall of symbols. The results of the Brown–Peterson Task reveal improved attention with large increases in the correct number of responses in the 18-s delay (+6) and the 36-s delay (+7) when compared with normative data for the 18-s delay (+0.44) and the 36-s delay (−0.1). There is sparse literature regarding music based cognitive rehabilitation and a gap in the literature between experimental research and clinical work. The purpose of this paper is to present the theory for Therapeutic Music Training (TMT) and to provide a pilot case study investigating the potential efficacy of TMT to remediate cognitive impairment following an ABI.

## 1. Introduction

An acquired brain injury (ABI) can result in impairment in a variety of domains including motor, speech, emotional, and cognitive. Cognitive impairment is the most common sequelae following an ABI [1,2,3,4] and is a result of deficit in one or more areas of cognition such as the various forms of attention, working memory, memory, executive function, or processing speed [5,6,7,8,9,10,11]. An individual with cognitive impairment may experience challenge to suppress distraction, remain on task, shift between tasks, follow directions, organize and initiate a response, or have difficulties with memory. Cognitive impairment can impact participation and progress in rehabilitation therapies for any of the above domains due to reduced attention, poor executive functioning, or impaired memory. The inability to attend to instructions of the therapist, to cognitively plan and organize a response, or to remember rehabilitation objectives outside the therapy session can potentially disqualify an individual from participation in rehabilitative programs or may impede progress in them. Furthermore, cognitive impairment is reported by family and caregivers as a significant source of stress [8,12,13,14]. Addressing cognitive impairment should be a priority in patient treatment following an acquired brain injury. Therefore, it is important to have on-going research into potentially effective cognitive rehabilitation tools.

Music training has been noted in the literature to impact areas of non-musical functioning including phonological awareness [15], speech processing [16], listening skills [17], perceiving speech in noise [18] and reading [19,20]. Of significance to the theory of Therapeutic Music Training, the literature demonstrates the impact of music training on cognitive abilities including attention and executive functioning [21,22,23,24,25,26,27].

Therapeutic Music Training (TMT) is a music therapy model in which the use of music training, specifically learning to play the piano, is used to address and remediate cognitive impairment following an acquired brain injury [28]. TMT is informed by clinical work and is grounded in literature. The hypothesis of the efficacy of TMT to remediate cognitive impairment is supported by literature regarding the influence of music training on cognition [23,24,25,29], musician’s enhanced abilities in attention, working memory, and cognitive control [26], theories of attention [30,31,32,33,34,35] and the neuroplasticity of the brain, including following injury [36,37,38,39,40]. Because of the engagement of the prefrontal cortex and the demands placed on working memory and attention during TMT, it can be an effective tool to address cognitive impairment. Although functionally interconnected, specific aspects of cognition such as working memory, attention, executive function, and memory are targeted in TMT tasks. TMT is a remedial approach to cognitive rehabilitation, that is, the goal is to drive, strengthen, and improve the underlying neural processes involved in the target cognitive areas. This is in contrast to a compensatory approach to cognitive rehabilitation, in which the goal is to provide the individual with strategies and accommodations to deal with the outcomes of cognitive impairment. The tangible outcome of producing a song provides motivation for the client to engage in cognitive rehabilitation and to remain in the rehabilitative process for an extended period of time as is required to stimulate a neuroplastic response and for the remediation of neural processing to take place.

TMT is distinct from modified music education in that the goal of TMT is the remediation of cognitive processes rather than music performance. Tasks involved in learning to play the piano are designed with the goal of placing demands on the various components of cognition. The sequencing and pacing of tasks are determined by the cognitive goals with consideration to target cognitive processes and the time required to drive and strengthen the networks involved. Novelty and the gradual increase in complexity of tasks are utilized to place on-going demands on attention networks and to gradually benefit higher cognitive processes. This is in contrast to modified music education, in which the primary goal is the acquisition of musical abilities and performance.

TMT is distinct from other models of music therapy in that it uses music training as the intervention for rehabilitative purposes. TMT contrasts from other music therapy models which use music primarily for expressive purposes, lack corrective feedback from the therapist, or use isolated music tasks which are not intended as music training. TMT is distinct from Neurologic Music Therapy (NMT) [41] in addressing cognitive goals as NMT does not use music *training* in its music-based rehabilitative interventions. Bruscia highlighted the importance of the music therapist’s “non-judgemental acceptance of what the client does musically” [42] (p. 3). While the TMT therapist would express empathy and support to the client, s/he would also provide constructive and corrective feedback as required in the learning to play an instrument. As in other models of music therapy, the therapist’s use-of-self and the role of the client–therapist relationship are important contributors to the success of the therapy.

Remarkably, much of cognitive rehabilitation is not grounded in the literature [36,43,44,45]. This may be due in part to the fact that rehabilitation therapy used to address cognitive impairment is most often based on a compensatory approach, accommodating or supporting the impairment, rather than attempting to remediate the cognitive processes that have been impaired. While the use of music and instrument playing for motor rehabilitation has been widely investigated [41,46,47,48], there is sparse literature investigating the potential efficacy of music-based cognitive rehabilitation interventions. This paper provides a brief introduction to the theory for TMT. This case study investigates the hypothesis of the potential effectiveness of therapeutic music training, TMT, to remediate cognitive impairment and serves as a pilot project to inform future, more rigorous studies. This investigation can contribute to the literature regarding music-based cognitive rehabilitation and inform clinical practice. There is a gap between cognitive experimental research and treatment applications [49]. The hypothesis for TMT has been informed by clinical work and this study can help fill in the gap between experimental research and clinical application.

### 1.1. The Role of the Prefrontal Cortex in Cognitive Rehabilitation

The prefrontal cortex (PFC), with widespread connections throughout the brain, is connected to sensory neocortical, motor systems, and subcortical structures [33]. As result of this widespread connectivity, the PFC is involved in several aspects of cognition and goal-directed behavior [45,50,51]. A key role of the PFC in cognition is its involvement with executive functioning: the ability to plan, organize, and initiate [45,52,53,54]. Miller [33] proposed the widespread connections of the PFC enable it to bias attention, memory, and motor responses to a common theme or target. This bias supports sustained attention to the target and the ability to organize a response. Following an acquired brain injury, an individual often demonstrates a lack of cognitive control, evident in the difficulty to sustain attention and remain on task, or to respond appropriately [55,56]. Lack of cognitive control, or a marked reduction in attentional abilities, is a common complaint post-brain injury. Because of its role in cognitive control and attention, remedial cognitive rehabilitation tasks should place demands on the PFC.

The literature supports the neuroplasticity of the PFC and that it is molded through training [35]. Repeated engagement in tasks that place demands on the PFC can improve and strengthen its connections. These tasks include those which require target selection, specific behavioral response, and the monitoring of errors. Tasks should place demands on the processes of working memory, attention, memory, and executive function.

### 1.2. The Role of Attention in Cognitive Rehabilitation

While attention, memory, and executive function are often divided in the literature, they are functionally interconnected. However, it is important to note that attention is foundational to memory and executive function. Improvements in attention processes can support improvement in other cognitive process [57,58,59,60]. Without attention, one cannot attend to a target, focus on important stimuli, remain on task, or consolidate memory. Because of the foundational role of attention in higher cognitive processes, it should be a key target in cognitive rehabilitation. Working memory and attention functionally overlap [32], thus tasks engaging working memory may also serve to strengthen attention processes.

### 1.3. TMT Engages the PFC and Attentional Processes

Chen et al. [45] stated that driving the re-organization of the PFC should be a primary goal of cognitive rehabilitation. This re-organization, or neuroplastic response, of the PFC is stimulated through repeated experience [33,61,62]. TMT engages the PFC through tasks that are involved in music training, tasks that include target selection and behavioral response, and tasks that engage attention, working memory, and executive functioning. The required review and practice of tasks involved in music training provides opportunity to repeatedly drive the cognitive processes engaged, thereby potentially strengthening these processes and cognitive networks, stimulating re-organization.

Based on theories of cognition and attention, TMT tasks have been developed to include the following criteria:

1. The intervention is designed to stimulate top-down processing to engage the PFC.
  - Detection and response to a target stimulus to place demands on attentional processing and to engage cognitive control networks
  - Goal-directed behavior
  - Effortful processing
2. The intervention places demands on working memory.
3. The intervention is designed to target a specific aspect of cognition informed by models of attention identified by Sohlberg and Mateer [63]. These include focused, sustained, selective, and alternating attention.
4. Interventions are administered following the hierarchy of attention and cognition, beginning with the level appropriate to the client.
5. Interventions are shaped throughout the treatment period, based on client progress, to gradually increase in complexity, and include novelty to ensure on-going engagement and stimulation of attentional processes. [61,63]. This may also support generalization of cognitive gains to ADLs.
6. The interventions are varied, highlighting melody, rhythm, or harmony and focusing on various senses such as sight, hearing, and motor to continue to engage and drive attention processes and prevent the acquisition of a “trained task” within a specific activity type, and thereby reducing the requirement for attentional effort.
7. Interventions are administered with consideration to the intensity and frequency of treatment, recognizing that a neuroplastic response is stimulated through experience.

The piano is the instrument used in TMT because the training can be adapted to the use of one hand or both, depending on any motor impairment the individual may experience in the hand or arm. Because music composed for the piano involves two clefs, this provides opportunity for engagement of alternating attention, an increased cognitive load, and a vast hierarchy of potential tasks to ensure on-going attentional demands and novelty throughout the training period.

Learning to decode notation, select the appropriate piano key(s), and evaluate the accuracy of the pitch and duration of the note(s) engages the PFC by placing demands on target selection, organized behavioral response, and monitoring of error. Reading note duration value (rhythm) also engages these cognitive networks. Playing a line of music engages working memory: short-term maintenance of the note’s name and location of the correct piano key; sustained attention: visually tracking from one note to the next note; long-term memory: recall of notes previously learned; alternating attention: reading two clefs; and focused attention: suppressing distraction from the environment or within the music itself in order to remain on task and to execute the line(s) of music as written. Throughout the treatment period, the TMT therapist must consider the cognitive goal, which music training tasks will place demands on the appropriate cognitive networks, and how to gradually shape the tasks and the music training assignments to continuously engage and strengthen those networks.

The assigned TMT homework given to the client provides opportunity for the driving of the cognitive networks between sessions, supporting the repetition and training required for a neuroplastic response and the strengthening of these networks. In addition to the cognitive processes involved in TMT, learning to play an instrument is a multi-model experience. The engagement of several senses such as vision, auditory, and motor may serve as a greater stimulus or support the learning process of an individual with an acquired brain injury who may be experiencing impairment in one or more modalities.

### 1.4. Previous Investigations into Music-Based Cognitive Rehabilitation

The potential cognitive benefits of music-based interventions have been acknowledged and investigated in previous studies [64,65,66,67]. Knox and Jutai [65] were pioneers investigating the potential benefit of music-based cognitive rehabilitation. They explored the cognitive domains engaged in the neural processing of music, proposed basing the design of music-based cognitive rehabilitation interventions on Sohlberg and Mateer’s [63] models of attention, and recommended the use of standardized neuropsychological tests to measure benefit of treatment. Knox, R., Yokota-Adachi, H., Kershner, J., and Jutai, J. [66] investigated the use of music-based training to address attention deficit in five adolescents following an acquired brain injury. The authors developed music-based attention tasks, using pre-recorded music, based on Sohlberg and Mateer’s [63] Attention Processing Training (APT) model. Their six-week study revealed success with a range of improvements between participants on sustained and selective attention tasks, while tasks targeting alternating and divided attention were noted as more difficult, with less improvement.

Moreno S. et al. [25] investigated the cognitive benefit of short-term music training and the generalization of benefit to non-music tasks. Their 20-day study compared the results of a visual arts group and a music-training group, each made up of children aged 4–6. Both groups used an interactive computer-based program. Their results indicated that only the music group demonstrated significant improvements on pre–post-test verbal scores, indicating transfer of benefit to non-music domain. The authors proposed this improvement was mediated through enhanced attention and memory rather than verbal ability. The music group also showed significantly higher gains in no-go trials suggesting improved executive functioning.

## 2. Case Study

The purpose of this case study was to investigate the potential effectiveness of TMT to engage and drive attentional processes, thereby improving the cognitive networks involved and increasing attentional abilities of the individual who has experienced cognitive impairment due to an acquired brain injury, and to inform a future, more rigorous study. Earlier clinical work of the author with several clients supported and informed the development of the hypothesis for TMT. This study was created, using standardized neuropsychological measures for pre- and post-treatment tests, to formally investigate TMT and its effectiveness. The case study, *A single subject pre-test–post-test study to investigate the effectiveness of Therapeutic Music Training to remediate attention and executive functioning impairments following an acquired brain injury*, received ethical approval from Wilfrid Laurier University (REB#5866, 16 January 2019).

The participant was recruited using purposeful sampling, specifically criterion sampling due to the participant meeting pre-determined criteria of importance, through an invitation letter which described the purpose of the investigation. The following were the criteria for inclusion in the study: (1) age ≥ 18; (2) has experienced an acquired brain injury; (3) has been diagnosed with cognitive impairment following an ABI with no known pre-existing cognitive deficit; (4) has the ability to complete the pre-and post-tests independently; (5) will not be receiving any other form of cognitive rehabilitation and (6) be a minimum of two years post-injury. The participant was a recent music therapy client of the investigator. The dual role of researcher–therapist was described and the invitation letter stated that any decision not to participate or to withdraw from the study would not impact the receiving of TMT. The participant signed a written consent form prior to the study.

### 2.1. Client Background

B.J. is an adult male, aged 56. Seven years ago, he was involved in a motor-vehicle accident (MVA) while waiting at an intersection for a red light. His vehicle was struck from behind at 80 km/h by a distracted driver and was pushed into the intersection where he collided into the vehicle in front of him. He sustained an ABI as a result of the accident. His cognitive abilities were assessed by a neurologist and test results revealed impairment in attention, memory, and executive functioning. Due to the level of impact of the ABI on various domains, the injury was identified as a catastrophic ABI. He was later also diagnosed with Post-Traumatic Stress Disorder (PTSD).

Prior to the MVA, B.J. was the Chief Executive Officer (CEO), the highest-ranking executive, of a high-tech company in a major city where he excelled in his work. He had excellent organizational skills and successfully multi-tasked throughout the typical workday. He was equally successful with technology and the interpersonal aspects of the company. He mentally calculated multi-million-dollar math equations. Post-ABI, B.J. could no longer count backwards or do mental math.

After the collision and for the following six years, B.J. experienced chronic neck and back pain, regularly occurring headaches, and severe migraines. He also experienced cognitive deficits of reduced attention, slowed cognitive processing, impaired memory, poor executive functioning, and word-finding challenges. He attempted to resume his career, but cognitive deficits, cognitive fatigue, and persistent headaches resulted in him being requested to retire from his position. He was unable to engage in any cognitively demanding task for more than 15–20 min, at which time a headache would typically be triggered. Cognitive tasks limited to 15–20 min included attending a meeting and tracking information, reading, writing, or watching a movie. B.J. stated he was unable to read more than one page of a book or magazine and could only complete simple addition and subtraction math equations. B.J. describes himself post-MVA as being “ADHD to the limit”, unable to remain focused on a task. Two years post-MVA, with minimal symptom improvement, B.J. began to also deal with depression. To address his symptoms, since the MVA, B.J. has been receiving case management, physiotherapy, occupational therapy, and psychotherapy. In Year 6, he also began receiving music therapy, specifically TMT.

The month prior to commencing TMT, B.J. received back surgery to address pain related to the MVA. This reduced neck and back pain and increased sensation in hands and feet. However, chronic migraine headaches remained. At the beginning of TMT, B.J. was demonstrating cognitive deficits to the same degree that he had been in the previous six years and continued to experience word-finding difficulties. He was referred to TMT by his occupational therapist to address cognitive impairment and to provide an opportunity to engage in music, an activity of personal significance, which could serve to reduce depressive symptoms.

### 2.2. Methodology

This study was approved by the ethics review committee of Wilfrid Laurier University. The participant was recruited through an invitation letter describing the study and the dual-role of therapist–researcher. The participant signed a consent form prior to the commencement of the study. The participant had received nine sessions, over nine months, prior to the formal commencement of the study and the administration of pre-tests. Session continuity of the pre-treatment sessions was interrupted due to a family emergency of the therapist and regular migraine headaches for the participant, which resulted in cancelled sessions.

TMT sessions initially focused on sustained and focused attention. Information was presented and practiced in 10–15-min blocks followed by a break. This allowed the participant to remain on task and acquire information, but not experience cognitive fatigue or to trigger a headache. Information was provided in small amounts to support attention and successful memory retention. Note identification was initially limited to five notes on a single (treble) clef and music was limited to one or two lines (4–8 bars). As information was successfully retained, novelty and increased complexity of information were introduced. This ensured on-going engagement of attention. New cognitive tasks were shaped and paced according to participant’s cognitive abilities and included new notes in the bass clef, alternating reading of both treble and bass clef, simultaneous reading of treble and bass clef, and varied rhythmic patterns. Music reading increased to a maximum of 32 bars. However, when reading 32 bars, cognitive fatigue often became evident after approximately 16 bars. Therefore, this task was balanced with shorter and varied tasks within the session and a break. At the end of treatment, the participant was beginning to read harmonic intervals, more than one note at a time, per clef. Selective attention was targeted in the final two months of treatment. The therapist played duet accompaniment, requiring the participant to remain focused on his own music while simultaneously hearing new and distracting music. Working memory was inherently engaged in the de-coding of notes and their execution on the keyboard.

### 2.3. Pre–Post Tests

Three standardized neuropsychology tests were used to measure components of attention: Trail Making Test, Digit Symbol Test, and the Brown–Peterson Task (auditory consonant trigram). The pre-tests and post-tests were administered by two individuals external to the study who were trained in test administration. Post-tests were administered after a 12-month treatment period.

#### 2.3.1. Trail Making Test

The Trail Making Test is a measure of attention, cognitive speed, and flexibility. Trail Making Test includes two separate tests: Trail Making A and Trail Making B. Trail Making A requires the participant, using a pencil, to connect alphabet letters in the proper order, which are presented randomly on the page. Trail Making B requires the individual to alternatingly connect letters and numbers in the proper order, which are presented randomly on the page. Scoring is based on time to complete the task. To ensure the participant understands the test requirements, a short practice test version is completed prior to the test.

Trail Making A is sensitive to maintenance of attention. Trail Making B places greater cognitive demands on visual search, cognitive flexibility, and motor speed due to the shifting of attention between letters and numbers, increased line length, and increase of items within 3-cm distance [68]

Trail Making Test is a standardized neuropsychological test [69,70,71,72] ranked as the top instrument to measure attention and fourth in use for measuring executive functioning [73].

#### 2.3.2. Digit Symbol Test

The Digit Symbol Test is used to test divided attention [74]. It also places demands on visual scanning, perceptual speed, motor speed, and memory [75,76]. The version of the Digit Symbol Test used in this case study also included a measurement of incidental learning [77].

The Digit Symbol Test requires the participant to check a symbol key where each symbol is identified with a number and, in the following chart, fill in the correct symbol that corresponds to the number provided. The numbers in this chart are in non-sequential order. To measure incidental learning, the participant is required to fill in a second chart with the appropriate symbol to the numbers provided without the symbol key available for reference. The final step of the Digit Symbol Test is for the participant to write on a blank page any symbols they recall, regardless of number. Scoring of the Digit Symbol Test includes identifying location on the first graph at two minutes, the number of correct symbols recalled in incidental learning and the number of correct symbols in free-recall.

The Digit Symbol is a standardized neuropsychological test most often used to assess divided attention, visual scanning, and motor speed [78,79,80,81,82,83].

#### 2.3.3. Brown–Peterson Task

The Brown–Peterson Task [84] measures working memory, the maintenance of information during distraction. Lower performance is observed with individuals with memory, attention, or executive function deficits [85]. The version used in this study was the Auditory Consonant Trigrams [71] in which the participant is required to remember three letters after varied lengths of delays. Delays were 9, 18, and 36 s long. During the delay the participant is required to do mental math. Scoring is based on the number of correct letters recalled following the delay.

The Brown–Peterson Task is a standardized neuropsychological assessment mostly used to measure working memory [71,86,87,88,89,90].

### 2.4. Results

#### 2.4.1. Trail Making A and B

The results of the Trail Making A reveal a marked decrease (−26.88) in test times (Table 1). The results of Trail Making B also reveal a large decrease (−20.30). Comparison of the participant’s results to normative data on both Trail Making A (−0.96) and Trail Making B (−3.86) indicate that participant decreased his post-test times by a much larger interval.

#### 2.4.2. Digit Symbol Test

The results of the Digit Symbol test did not reveal any change in incidental learning with the correct recall of three symbols in both the pre- and post-tests (Table 2). The results reveal a very small increase in speed of processing (+1 block) in location on test at two minutes. Post-test results indicate a reduction (−2.0) in the number of correct symbols remembered in free-recall.

#### 2.4.3. Brown–Peterson Task

Comparison of pre- and post-tests of the Brown–Peterson task indicate a slight decrease of correct responses (−1) on the 9-s delay (Table 3). However, the number of correct responses increased markedly for the 18-s delay (+6) and the 36-s delay (+7). This is in contrast to normative data, which reveal a very small improvement on the 9-s delay (+0.23), 18-s delay (+0.44), and 36-s delay (+0.1).

### 2.5. Anecdotal Evidence

In addition to post-test results, observations made by the participant, his family members, and the therapist also supported improving cognitive functioning as a result of treatment. Over the course of treatment, the participant’s attention span increased during TMT sessions from 15–20 min of cognitive tasks before cognitive fatigue resulted in the need for a break to 30–40 min. This increased attention span also became evident in between-session homework practice, which increased from 15 to 30–40 min. After the first three months of treatment, the participant’s family reported that his ability to attend and remain on task generalized and was maintained for approximately 2 h post session. Interestingly, this improvement coincided with a reduction in word-finding problems and reduced pauses in sentence fluency. This reduction in word-finding difficulties was also observed by the friends of the participant in social settings and by the therapist during sessions. Generalization of improved attention was also demonstrated in the increased time from 15 to 30 min for reading.

## 3. Discussion

The purpose of this pilot study was to determine if there was evidence of effect of treatment, specifically TMT, on the cognitive performance of an individual with an acquired brain injury. If treatment effect were indicated, this study could support the theory of Therapeutic Music Training, serve to expand clinical practice by providing a music-based cognitive rehabilitation model, and inform future research and a more rigorous study.

### 3.1. Results Support the Theory for the Efficacy of TMT

#### 3.1.1. Trail Making A and B

The results of both Trail Making A and Trail Making B point favorably to treatment increasing the maintenance of attention and cognitive speed. The participant notably decreased his test times, completing Trail Making A post-test 203.79% faster and Trail Making post-test B 138.22% faster. In comparison to normative data in which individuals did not receive treatment, the participant made large improvements. Trail Making Test is ranked top in measuring attention and fourth in measuring executive functioning and therefore is reliable to use in assessing that the participant has improved his attentional abilities following treatment. These results support the hypothesis for TMT to engage attentional processes and to improve attention. Trail Making A and B post-tests both reveal a decrease in test time, indicating improved attention and increased processing speed.

#### 3.1.2. Digit Symbol

The results of the Digit Symbol test did not reveal any positive changes and free recall demonstrated a reduction (−2) in symbols remembered. This may indicate a lack of improvement in divided attention. This result is also reflected in another study investigating music-based cognitive rehabilitation [66] in which divided attention tasks were described as more difficult and measures did not reveal improvement while other forms of attention did. Depending on the ABI lesion site(s) and other factors, one form of attention may be more difficult to remediate than another or may require more treatment time. Another important consideration for lack of results on the Digit Symbol is the reliance on memory. The TMT treatment did not focus on memory but instead on attentional processes and working memory.

#### 3.1.3. Brown–Peterson Task

Brown–Peterson Task is the most widely used measure of working memory. Participant results indicate a small reduction (−1) in correct responses following a 9-s delay. However, there was a marked improvement on correct responses following 18- and 36-s delays. These results further support the improvements noted in the Trail Making Test as working memory and attention functionally overlap [32]. The improvements noted in the Brown–Peterson task (working memory) could be expected to impact the results of Trail Making Test (attention), providing support to an overall improvement in attentional processing following TMT treatment.

The participant’s greater interval of improvement in comparison to normative data on Trail Making A and the Brown–Peterson Task may be attributed to the participant’s level of cognitive impairment and therefore broader range for potential improvement.

The marked improvements in post-test results Trail Making A and B and the Brown–Peterson Task reflect improvement in attention, working memory, and executive functioning. These results support the theory of the efficacy of TMT to engage the target cognitive networks and to improve functioning. The treatment period of 12 months provided opportunity for the repeated driving of attentional processes and the improvement of these networks. The results of the Digit Symbol test reveal that this participant did not improve in divided attention. More treatment time may be required to observe results or this form of attention may be more difficult to remediate for this individual. Due to the results of this study and those of Knox et al. [66], future studies might investigate and compare results of remediation of various forms of attention.

Prior to TMT, the participant had not made notable progress in cognitive rehabilitation in the previous six years. This study demonstrates the effectiveness of TMT to address cognitive rehabilitation goals, specifically attention and working memory, for an individual who has sustained an ABI. TMT was effective six years post injury, several years after spontaneous recovery may have occurred. The apparent transfer of benefit of TMT may be supported in part by the variety of the cognitive tasks within TMT and by the length of treatment period.

### 3.2. This Case Study’s Results Support Other Findings

The post-test results demonstrating the efficacy of TMT to address cognitive rehabilitation goals support other findings in the literature. Learning has been identified as a stimulus for neuroplastic change [36,43,46,90,91,92,93,94,95] which supports the rationale to use the experience of learning new information and a skill to drive a neuroplastic response. The literature demonstrates the potential for cognitive gains through cognitive rehabilitation [58,61,64,96,97,98,99,100,101,102,103,104], supporting the theory for remediation of cognitive impairment through specific cognitive tasks. The use of music-based cognitive tasks, and in particular the learning to play an instrument to target cognitive goals, is supported by numerous studies demonstrating musicians’ enhanced executive functioning and the benefit of this enhancement on cognitive processes [22,23,24,25,26,27,104]. Furthermore, the literature also demonstrates musicians’ recruitment of larger neural networks involved in cognitive control and sustained attention when engaged in a difficult memory task [22,26]. The rationale for the theory of the efficacy of TMT is well supported in the literature, demonstrating that cognitive remediation following brain trauma is possible and that music in particular may provide a unique and powerful stimulus for cognitive networks. Moreno and Bidelman [67] stated that “music’s impact on the brain is unique in that it offers distinct perceptual and cognitive benefits not observed with other forms of intense training or experience. Perhaps more significantly, music training is a rare activity that modifies a hierarchy of brain structures” (p. 93). TMT not only provides cognitive tasks that place demands on attentional processes, but it uses *music* tasks, providing a unique form of cognitive demand and stimulus to drive and strengthen cognitive networks and improve cognitive functioning post ABI.

The results of this study demonstrating cognitive benefit from music training support the findings of other studies investigating the brain’s neuroplastic response to music training, specifically learning to play an instrument. Bugos et al. [105] found significantly improved performance on cognitive tests measuring executive functioning and working memory in senior adults aged 60–85 following six months of piano lessons. These results were also reflected by Seinfeld et al. [106] following four months of daily piano training. 

Stewart et al. [107] revealed neuroplastic changes in response to reading music notation and in motor response after 15 weeks of piano lessons. Fujioka et al. [108] observed enhanced auditory perception in children following 12 months of violin lessons. Fujioka et al. [108] also demonstrated improved performance on a cognitive measure, Digit Span, suggesting improved working memory and attention following music training. 

While several studies have investigated the impact of music or music training on cognitive abilities, very few studies have explored the use of music for cognitive rehabilitation following ABI. The results from earlier studies investigating the potential of music-based cognitive rehabilitation following ABI are reflected in the results of this case study. Knox et al. [66] stated that music-based cognitive tasks resulted in improved sustained and selective attention tasks, while alternating and divided attention were noted as being more difficult and with less improvement. The post-test results of this study reflect similar findings, with sustained attention showing improvement while the Digit-Symbol test measuring alternating attention did reveal improvement. This may be due to a longer treatment time needed in order for gains to be observed in alternating attention or may be due to the brain injury site(s) involved.

While these early studies demonstrate the effectiveness of music to engage and improve attention, TMT is unique in that it is a treatment model that includes learning to read music and the active participation of playing an instrument rather than a computer-based music training program. Within TMT, there is a wide range of potential cognitive tasks involved in the process of learning to read music and play an instrument that can be structured and adapted to the individual’s need and pace, providing an on-going hierarchy of cognitive demand and the driving of attentional processes The engagement of multisensory involvement in playing an instrument can serve to be a stronger and multi-site stimulus for the injured brain. 

Following music training, Moreno et al. [26] observed improvements measured on verbal scores and attributed these gains to increased attention and memory rather than verbal ability. Interestingly, while not a goal area, as attention abilities began to improve, the participant in this study demonstrated increased word fluency and a reduction in word-finding problems during the treatment period. 

An important aspect of TMT is the intrinsic motivation involved when learning to play an instrument. McPherson and O’Neil [109] highlighted the motivation that learning music inspires. This motivation is important in order for the individual to remain engaged in the rehabilitation process for the time required to gain benefit. The journey of rehabilitation is often lengthy and challenging. Individuals with ABI may lack the insight to commit to the therapeutic process. They may become discouraged during rehabilitation. TMT, with the rewarding and tangible outcome of producing a song, can provide the needed motivation to remain in therapy for the period of time required to observe gains. The positive emotions and reduced anxiety and agitation associated with music was proposed by Peck et al. [110] to support enhanced attention 

The findings of this study are reflected in other studies exploring the potential for cognitive gains as a result of music training, including in individuals with cognitive impairment following an ABI. The results of this case study favorably point to the potential effectiveness of TMT as a cognitive rehabilitation tool to remediate cognitive impairment following an acquired brain injury, however more research is required, optimally with control groups and experimental design. 

## 4. Limitations

### 4.1. Single Subject Study Design

Although the post-test results point favorably to treatment effect, the absence of a control subject prevented statistical analysis for significance. The one-point data of this study results in limitations to the interpretation of the impact or generalization of results.

### 4.2. Length of Treatment Period

The length of treatment might be considered a limitation to this study as treatment was provided for 12 months prior to post-tests. A contributing factor in the length of treatment was the high number of session cancellations due to the participant’s frequent migraine headaches. Migraine headaches and other health challenges are a reality for many individuals living with the outcomes of an ABI and the treatment period for future studies will need to accommodate this issue when facilitating investigations with this clinical population. Although in-person treatment sessions were regularly cancelled, the participant continued to practice TMT homework between sessions, thereby maintaining the required cognitive stimulation and the driving of the PFC and cognitive networks.

The 12-month treatment period allowed for repeated driving of cognitive processing, supporting the strengthening of attention and memory processes. Furthermore, the 12-month treatment between pre-and post-tests could serve to reduce any potential “practice effect” of tests.

## 5. Future Research Directions

Future research investigating the efficacy of TMT should be designed to include an experimental group with an ABI receiving treatment and a control group with ABI that is not receiving treatment. This would allow for multi-data point comparison of the effectiveness and potential statistical significance of treatment.

To determine minimal treatment time required for improvement or to capture increasing cognitive gains, future studies should include multiple test points within the treatment period. A neuroplastic response requires experience and repetition. This study had a 12-month treatment period to accommodate for migraine-related cancelled sessions and to provide enough time for repeated task-driving of cognitive networks to strength these networks. Future studies should include assessment at 3-, 6-, or 9-month treatment points to determine the earliest treatment point that demonstrates improvement or to investigate for an optimal point of progress within the treatment period.

Future studies could investigate the potential link between improved attention abilities and improved speech measures.

Future studies could investigate and compare the results of the remediation of the various forms of attention.

While the patient indicated improved mood and self-esteem during TMT, this theme could be explored in future studies.

## Figures and Tables

**Table 1:** Results of Trail Making Test A and B and Comparison to Normative Data

Trail Making Test	Participant			Norms Adults. Normal or Neurologically Stable (*n* = 384)		
	Pre-Test	Post-Test	T2-T1	Test 1	Test 2	T2-T1
Test A	54.78	27.9	−26.88	26.52	25.56	−0.96
Test B	76.13	55.8	−20.33	72.05	68.19	−3.86

**Table 2:** Results of Digit Symbol Test and Comparison to Normative Data

Digit Symbol Test	Participant		Norms Age >50
	Pre-Test	Post-Test	
Incidental Learning	3	3	4.86
Free-recall	6	4	N/A

**Table 3:** Results of Brown-Peterson Task and Comparison to Normative Data.

Brown-Peterson Task	Participant			Norms Adults. Normal or Neurologically Stable. Age 50–69 (*n* = 30)		
	Pre-Test Number Correct/15	Post-Test Number Correct/15	T2-T1	Test 1 Number Correct/15	Test 2 Number Correct/15	T2-T1
9-s delay	15	14	−1	11.47	11.70	+0.23
18-s delay	7	13	+6	10.23	10.67	+0.44
36-s delay	3	10	+7	8.67	8.57	−0.1

## References

[1] Dikmen S.S., Machamer J.E., Winn H.R., Temkin N.R. (1995). Neuropsychological outcome at 1-year post head injury. Neuropsychology.

[2] Goldstein F.C., Levin H.S., Baddeley A.D., Wilson B.A., Watts F.N. (1996). Post-traumatic and anterograde amnesia following closed head injury. Handbook of Memory Disorders.

[3] Gronwall D., Levin H.S., Grafman J., Eisenberg H.M. (1987). Advances in the assessment of attention and information processing after head injury. Neurobehavioural Recovery from Head Injury.

[4] Van Zomeren A.H., Brouwer W.H. (1994). Clinical Neuropsychology of Attention.

[5] Beers S.R. (1987). Cognitive effects of mild head injury in children and adolescents. Neuropsychol. Rev..

[6] Donders J. (1993). Memory functioning after traumatic brain injury in children. Brain Injury.

[7] Kaufmann P.M., Fletcher J.M., Levin H.S., Miner M.E. (1993). Attentional disturbance after pediatric closed head injury. J. Child Neurol..

[8] Whyte E., Skidmore E., Aizenstein H., Richer J., Butters M. (2011). Cognitive impairment in acquired brain injury: A predictor of rehabilitation outcomes and an opportunity for novel interventions. Am. Acad. Phys. Med. Rehabil..

[9] Ballard C., Stephens S., Keeny R., Kalaria R., Tovee M., O’Brien J. (2003). Profile of neuropsychological deficits in older stroke survivors without dementia. Dement. Geriatr. Cogn. Disorder..

[10] Jaillard A., Naegele B., trabucco-Miguel S., LeBas J., Hommel M. (2009). Hidden dysfunctioning in subacute stroke. Stroke.

[11] Michel J.A., Mateer C.A. (2006). Attention rehabilitation following stroke and traumatic brain injury. Eur. Medicophys..

[12] Brooks N., McKinlay W., Symington C., Beattie A., Campsie L. (1987). Return to work within the first seven years of injury. Brain Inj..

[13] Kinsella G.I., Prior M., Sawyer M., Ong B., Murtagh D., Eisenmajor R., Bryan D., Anderson B., Klug G. (1997). Predictors and indicators of academic outcome in children 2 years following traumatic brain injury. J. Int. Neuropsychol. Soc..

[14] Van Zomeren A.H., Van Den Burg W. (1985). Residual complaints of patients two years after severe head injury. J. Neurol. Neurosurg. Psychiatry.

[15] Dege F., Kubicek C., Schwarezer G. (2011). Music lessons and intelligence: A relation mediated by executive functions. Music Percept..

[16] Wong P.C., Skoe E., Russo N.M., Dees T., Kraus N. (2007). Musical experience shapes human brainstem encoding of linguistic pitch patterns. Nat. Neurosci..

[17] Munte T.F., Kohlmetz C., Nager W., Altenmuller E. (2001). Superior auditory spatial tuning in conductors. Nature.

[18] Parberry-Clark A., Skoe E., Lam C., Kraus N. (2009). Musician enhancement for speech in noise. Ear Hear..

[19] Douglas S., Willatts P. (1994). The relationship between musical ability and literacy skills. J. Res. Read..

[20] Gardiner M.F., Fox A., Knowles F., Jeffery D. (1996). Learning improved by arts training. Nature.

[21] Gaab N., Schlaug G. (2003). The effect on musicianship on pitch memory in performance matched groups. Neuroreport.

[22] Hannon E., Trainor L. (2007). Music acquisition: Effects of enculturation and formal training on development. Trends Cogn. Sci..

[23] Bialystok E., DePape A.M. (2009). Musical expertise, bilingualism, and executive functioning. J. Exp. Psychol. Hum. Percept. Perform..

[24] Strait D.L., Kraus N., Parberry-Clark A., Ashley R. (2010). Musical experience shapes top-down auditory mechanisms: Evidence from masking and auditory attention performance. Hear. Res..

[25] Moreno S., Bialystok E., Barac R., Schellenberg E.G., Cepeda N.J., Chau T. (2011). Short-term music training enhances verbal intelligence and executive function. Psychol. Sci..

[26] Pallesen K.J., Brattico E., Bailey C.J., Korvenoja A., Koivisto J., Gjedde A., Carlson S. (2010). Cognitive control in auditory working memory is enhanced in musicians. PLoS ONE.

[27] Strait D.L., Kraus N. (2011). Playing music for a smarter ear: Cognitive, perceptual, and neurobiological evidence. Music Percept..

[28] Jones C. (2020). Therapeutic Music Training (TMT): A music therapy model using music training on an instrument to address therapeutic goals in the areas of cognition and psychosocial health. Approaches.

[29] Chan A.S., Ho Y.C., Cheung M.C. (1998). Music training improves verbal memory. Nature.

[30] Baddeley A., Stuss D.T., Knight R.T. (2002). Fractionating the central executive. Principles of Frontal Lobe Function.

[31] Baddeley A. (2012). Working memory: Theories, models, and controversies. Ann. Rev. Psychol..

[32] Corbetta M., Shulman G.L. (2002). Control of goal-directed and stimulus driven attention in the brain. Nat. Rev. Neurosci..

[33] Miller E.K. (2000). The prefrontal cortex and cognitive control. Nat. Rev. Neurosci..

[34] Miller E.K., Cohen J.D. (2001). An integrative theory of prefrontal cortex function. Annu. Rev. Neurosci..

[35] Peterson S.E., Posner M.I. (2012). The attentional system of the human brain: 20 years later. Annu. Rev. Neurosci..

[36] Bach-y-Rita P. (1992). Recovery from brain damage. J. Neuro. Rehab..

[37] Doidge N. (2015). The Brain’s Way of Healing.

[38] Duffau H. (2005). Lessons from brain mapping in surgery for low-grade glioma: Insights into associations between tumor and brain plasticity. Lancet Neurol..

[39] Duffau H. (2009). Does post-lesional subcortical plasticity exist in the human brain?. Neurosci. Res..

[40] Rossini P.M., Calautti C., Pauri R., Baron J.C. (2003). Post-stroke plastic reorganization in the adult brain. Lancet Neurol..

[41] Thaut M.H., McIntosh G.C., Hoemberg V., Thaut M., Hoemberg V. (2014). Neurologic Music Therapy: From social science to neuroscience. The Handbook of Neurologic Music Therapy.

[42] Bruscia K. (1998). Defining Music Therapy.

[43] Mateer C.A., Kerns K.A. (2000). Capitalizing on neuroplasticity. Brain Cogn..

[44] Ceravolo M.G. (2006). Cognitive rehabilitation of attention deficit after brain damage: From research to clinical practice. Eur. Medicophys..

[45] Chen A.J.W., Abams G.M., D’Esposito M. (2006). Functional reintegration of prefrontal neural networks for enhancing recovery after brain injury. J. Head Trauma Rehabil..

[46] Altenmuller E., Marco-Pallares J., Munte T.F., Schneider S. (2009). Neural reorganization underlies improvement in stroke-induced motor dysfunction by music-supported therapy. Neurosci. Music III Disord. Plast Ann. N. Y. Acad. Sci..

[47] Rodriguez-Fornells A., Rojo N., Amengual J.L., Ripolles P., Altenmuller E., Munte T.F. (2012). The involvement of audio-motor coupling in the music-supported therapy applied to stroke patients. Ann. N. Y. Acad. Sci. Neurosci. Music IV Learn. Mem..

[48] Fujioka T., Ween J.E., Jamali S., Stuss D.T., Ross B. (2012). Changes in neuromagnetic beta-band oscillation after music-supported stroke rehabilitation. Ann. N. Y. Acad. Sci. Neurosci. Music IV Learn. Mem..

[49] Burgess P.W., Robertson I.H., Stuss D.R., Knight R.T. (2002). Principles of the rehabilitation of the frontal lobe function. Principles of Frontal Lobe Function.

[50] D’Esposito M., Postile B.R., Stuss D.T., Knight R.T. (2002). The organization of working memory function in lateral prefrontal cortex: Evidence from event-related functional MRI. Principles of Frontal Lobe Function.

[51] Cohen J.D., Braver T.S., O’Reilly R.C., Roberts A.C., Robbins T.W., Weiskrantz L. (1998). A computational approach to prefrontal cortex, cognitive control, and schizophrenia: Recent developments and current challenges. The Prefrontal Cortex: Executive and Cognitive Functions.

[52] Anderson V., Levin H.S., Jacobs R., Stuss D., Knight R. (2002). Executive functions after frontal lobe injury: A developmental perspective. Principles of Frontal Lobe Function.

[53] Krebs C., Weinberg J., Akesson E. (2012). Lippincott’s Illustrated Reviews: Neuroscience.

[54] Levine B., Robertson I.H., Clare L., Carter G., Hong J.U., Wilson B.A., Duncan J., Stuss D.T. (2000). Rehabilitation of executive function: An experimental-clinical validation of Goal Management Training. J. Int. Neuropsychol. Soc..

[55] Dockree P.M., Kelly S.P., Roche R.A.P., Hogan MJReilly R.B., Robertson I.H. (2004). Behavioural and physiological impairments of sustained attention after traumatic brain injury. Cogn. Brain Res..

[56] Chen J.W.A., Novakovic-Agopian Tatjana Nycum T.J., Song S., Turner G.R., Hills NKKRome SAbrams G.M., D’Esposito M. (2011). Training of goal-irected attention regulation enhances control over neural processing for individuals with brain injury. Brain.

[57] Mateer C.A., Sohlberg M.M., Whitaker H. (1988). A.; Sohlberg, M.M. A paradigm shift in memory rehabilitation. Neuropsychological Studies of Nonfocal Brain Injury: Dementia and Closed Head Injury.

[58] Mateer C.A., Sohlberg M.M., Youngman P., Wood R., Fussey I. (1990). The management of acquired attention and memory disorders following mild closed head injury. Cognitive Rehabilitation in Perspective.

[59] Mateer C.A., Horn L., Zasler N. (1992). Systems of care for post-concussive syndrome. Rehabilitation of Post-Concussive Disorders.

[60] Niemann H., Ruff R.M., Basser C.A. (1990). Computer-assisted attention training in head injured individuals: A controlled efficacy study in an outpatient group. J. Consult. Clin. Psychol..

[61] Kelly C., Foxe J.J., Garavan H. (2006). Patterns of normal brain plasticity after practice and their implications for neurorehabilitation. Arch. Phys. Med. Rehabil..

[62] O’Connell R.G., Robertson I.H., Raskin S. (2011). Plasticity of high-order cognition: A review of experience-induced remediation studies for executive deficits. Neuroplasticity and Rehabilitation.

[63] Sohlberg M.M., Mateer C.A. (2001). Cognitive Rehabilitation: An Integrative Neuropsychological Approach.

[64] George E.M., Coch D. (2011). Music training and working memory: An ERP study. Neuropsychologia.

[65] Knox R., Jutai J. (1996). Music-based rehabilitation of attention following brain injury. Can. J. Rehabil..

[66] Knox R., Yokota-Adachi H., Kershner J., Jutai J. (2003). Musical attention training program and alternating attention in brain injury: An initial report. Music Ther. Perspect..

[67] Moreno S., Bidelman G.M. (2014). Examining neural plasticity and cognitive benefit through the unique lens of musical training. Hear. Res..

[68] Stuss D.T., Eskes G.A., Foster J.K., Oller F.B., Grafman J. (1994). Experimental neuropsychological studies of frontal lobe functions. Handbook of Neuropsychology.

[69] Barr W.B. (2003). Neuropsychological testing of high school athletes: Preliminary norms and test-retest indices. Arch. Clin. Neuropsychol..

[70] Lucas J.A., Ivnik R.J., Smith G.E., Ferman T.J., Willis F.B., Petersen R.C., Graff-Radford N.R. (2005). Mayo’s Older African American Normative Stuides: Norms for Boston Naming Test, Controlled Oral Word Association, Category Fluency, Animal Naming, Token Test, WRAT-3 Reading, Trail Making Test, Stroop Test, and Judgment of Line Orientation. Clin. Neuropsychol..

[71] Mitrushina M.N., Boone K.B., Razani J., D’Elia L.F. (2005). Handbook of Normative Data for Neuropsychological Assessment.

[72] Tombaugh T.N. (2005). Trail Making Test A and B: Normative data stratified by age and education. Arch. Clin. Neuropsychol..

[73] Rabin L.A., Barr W.B., Burton L.A. (2005). Assessment practices of clinical neuropsychologists in United States and Canada: A survey of INS, NAN, and APA Division 40 members. Arch. Clin. Neuropsychol..

[74] Ponsford J.L., Kinsella G. (1988). Evaluation of a remedial programme for attentional deficits following closed head injury. J. Clin. Exp. Neuropsychol..

[75] Laux L.F., Lane D.M. (1985). Information processing components of substitution test performance. Intelligence.

[76] Lezak M.D. (1995). Neuropsychological Assessment.

[77] Uchiyama C.L., D’Elia L.F., Delinger A.M., Selnes O.A., Becker J.T., Wesch Je Chen B.B., Satz P., Van Gorp W., Miller E.N. (1994). Longitudinal comparison of alternate versions of the Symbol Digit Modalities Test: Issues of form comparability and moderating demographic variables. Clin. Neuropsychol..

[78] Bowler R., Sudia S., Mergler D., Harrison R., Cone J. (1992). Comparison of Digit Symbol and Symbol Digit Modalities Tests for assessing neurotoxic exposure. Clin. Neuropsychol..

[79] Emmerson R.Y., Dustman R.E., Shearer D.E., Turner C.W. (1990). P3 latency and symbol digit performance correlations in aging. Exp. Aging Res..

[80] Gilmore G.C., Royer F.L., Gruhn J.J. (1983). Age differences in symbol-digit substitution task performance. J. Clin. Psychol..

[81] Joy S., Kaplan E., Fein D. (2004). Speed and memory in the WAIS-III Digit Symbol-Coding subtest across the adult lifespan. Arch. Clin. Neuropsychol..

[82] Hinton-Bayre A.D., Geffen G. (2005). Comparability, reliability, and practice effects on alternate forms of the Digit Symbol and Symbol Digit Modalitites Tests. Psychol. Assess.

[83] Yeudall L.T., Fromm D., Reddon J.R., Stefanyk W.O. (1986). Normative data stratified by age and sex for 12 neuropsychological tests. J. Clin. Psychol..

[84] Peterson L.R., Peterson M.J. (1959). Short-term retention of individual verbal items. J. Exp. Psychol..

[85] Strauss E., Sherman E.M.S., Spreen O. (2006). A Compendium of Neuropsychological Tests: Administration, Norms, and Commentary.

[86] Bherer L., Belleville S., Petetz I. (2001). Education, age, and the Brown-Peterson technique. Dev. Neuropsychol..

[87] Boone K.B., Miller B.L., Cummings J.L. (1999). Neuropsychological assessment of executive functions: Impact of age, education, gender, intellectual level, and vascular status on executive test scores. The Human Frontal Lobes: Functions and Disorders.

[88] Floden D., Stuss D.T., Craik F.I.M. (2000). Age difference in performance on two versions of the Brown-Peterson task. Aging Neuropsychol. Cogn..

[89] Kopelman M.D., Stanhopes N. (1997). Rates of forgetting in organic amnesia following temporal lobe, diencephalic, or frontal lobe lesions. Neuropsychology.

[90] Draganski B., Gaser C., Busch V., Schuierer G., Bogdahn U., May A. (2004). Changes in grey matter induced by training. Nature.

[91] Lee Y., Lu M., Ko H. (2007). Effects of skill training on working memory capacity. Learn. Instr..

[92] Habib M., Besson M. (2009). What do music training and musical experience teach us about brain plasticity?. Music Percept..

[93] Pantev C. (2009). Music training and induced cortical plasticity. The neuroscience and music III: Disorders and plasticity. Ann. N. Y. Acad. Sci..

[94] Schlaug G., Forgeard M., Zhu L., Norton A., Norton A., Winner E. (2009). Training-induced neuroplasticity in young children. The Neurosciences and Music III: Disorders and Plasticity. Ann. N. Y. Acad. Sci..

[95] Trainor L., Shahim A.J., Roberts L.E. (2009). Understanding the benefits of musical training: Effects on oscillatory brain activity. The Neurosciences and Music III: Disorders and Plasticity.. Ann. N. Y. Acad. Sci..

[96] Gummow L., Miller P., Dustman R.E. (1983). Attention and brain injury: A case for cognitive rehabilitation of attentional deficits. Clin. Psychol. Rev..

[97] Mateer C.A., Mapou R.L. (1996). Understanding, evaluating, and managing attention disorders following traumatic brain injury. J. Head Rehabil..

[98] Van den Broek M.D. (1999). Cognitive rehabilitation and brain injury. Rev. Clin. Gerontol..

[99] Sohlberg M.M., Avery J., Kennedy M., Ylvisaker M., Coelho C., Turkstra L., Yorkston K. (2003). Practice guidelines for direct attention training. J. Med. Speech Lang. Pathol..

[100] Sturm W., Longoni F., Weis S., Specht K., Herzog H., Vohn R., Thimm M., Willmes K. (2004). Functional reorganization in patients with right hemisphere stroke after training of alertness: A longitudinal PET and fMRI study in eight cases. Neuropsychologia.

[101] Cicerone K., Levin H., Malec J., Stuss D., Whyte J. (2006). Cognitive rehabilitation interventions for executive function: Moving from bench to bedside in patient with traumatic brain injury. J. Cogn. Neurosci..

[102] Kim Y.H., Yoo W.K., Ko M.H., Park C.H., Kim S.T., Na D.L. (2009). Plasticity of attentional network after brain injury and cognitive rehabilitation. Neurorehabil. Neural Repair.

[103] Engle J.A., Kerns K.A., Raskin S. (2011). Neuroplasticity and rehabilitation of attention in children. Neuroplasticity and Rehabilitation.

[104] Barrett K.C., Ashley R., Strait D.L., Kraus N. (2013). Art and science: How musical training shapes the brain. Front. Psychol..

[105] Bugos J.A., Perlstein W.M., McCrae C.S., Brophy T.S., Bedenbaugh P.H. (2007). Individualized piano instruction enhances executive functioning and working memory in older adults. Aging Ment. Health.

[106] Seinfeld S., Figueroa H., Ortiz-Gil J., Sanchez-Vives M.V. (2013). Effects of learning and piano practice on cognitive function, mood and quality of life in older adults. Front. Psychol..

[107] Stewart L., Henson R., Kampe K., Walsh V., Turner R., Frith U. (2003). Brain changes after learning to read and play music. NeuroImage.

[108] Fujioka T., Ross B., Kakigi R., Pantev C., Trainor L.J. (2006). One year of musical training affects development of auditory cortical-evoked fields in young children. Brain.

[109] McPherson G.E., O’Neill S.A. (2010). Students’ motivation to study music as compared to other school subjects: A comparison of eight countries. Res. Stud. Music Educ..

[110] Peck K.J., Girard T.A., Russo F.A., Fiocco A.J. (2006). Music and memory in Alzheimer’s disease and the potential underlying mechanisms. J. Alzheimer’s Dis..

